# Effects of Bedding Material on Equine Lower Airway Inflammation: A Comparison of Two Peat Beddings, Wood Pellet, and Straw Pellet

**DOI:** 10.3389/fvets.2021.799645

**Published:** 2021-12-17

**Authors:** Jenni Mönki, Markku Saastamoinen, Ninja Karikoski, Marianna Norring, Minna Rajamäki, Anna Mykkänen

**Affiliations:** ^1^Department of Equine and Small Animal Medicine, University of Helsinki, Helsinki, Finland; ^2^Natural Resources Institute Finland, Jokioinen, Finland; ^3^Department of Production Animal Medicine, University of Helsinki, Helsinki, Finland

**Keywords:** equine airway inflammation, equine asthma, bedding material, horse, dust, stable air quality, bronchoalveolar lavage, environmental allergens

## Abstract

The effects of bedding material on air quality are important amongst horses worldwide. Respiratory diseases, especially equine asthma, are highly prevalent with air hygiene playing a major role on the pathophysiology of these diseases. The objective of our study was to investigate the effects of four bedding materials on the respiratory signs, tracheal mucus score, and tracheal wash (TW) and bronchoalveolar lavage fluid (BALF) cytology in healthy adult horses. The study design was a prospective controlled cross-over study, and the subjects were healthy adult riding school horses (*n* = 32) from a single stable. Wood pellet, straw pellet, and loosely stored peat (Peat 3) were compared to peat packed in plastic-covered bales (Peat 2). Lower airway endoscopy and sampling (TW and BALF) for cytological examination were performed after each 35-day bedding period. The tracheal mucus scores (*P* = 0.014) and respiratory rate (*P* = 0.026) were higher during the straw pellet period compared to the Peat 2 period. The respiratory rate was lower during the wood pellet period compared to the Peat 2 period (*P* = 0.004). The TW neutrophil percentage during the straw pellet period was higher compared to the Peat 2 period (*P* = 0.0003). The BALF neutrophil percentage was higher during the straw pellet period (*P* = 0.005) and during the Peat 3 period compared to the Peat 2 period (*P* = 0.04). We conclude that baled peat (Peat 2) caused lower neutrophil percentages in the airway samples compared to straw pellet and loosely stored peat (Peat 3). No difference was observed between Peat 2 and wood pellet. The information gained from this study may assist veterinarians and horse owners in selecting appropriate bedding materials, especially for horses with equine asthma.

## Introduction

Equine management systems often involve horses being housed inside for most of the day throughout their lives. The types of forage and bedding material used are the largest contributors to stable air quality, especially its dust concentrations (1–3). Exposure to inhaled dust and molds is considered one key element in the pathophysiology of equine asthma (4) and in the exacerbation of this disease (5, 6). On the other hand, when the number of environmental allergens is controlled, asthmatic horses may go into remission (3).

Straw and wood shavings have historically been commonly used as horse bedding. Wood pellets and straw pellets have increased their popularity due to their practicality: smaller volumes of fresh bedding are needed, as these pellets expand after absorbing some moisture, and mucking may be less time-consuming than with more traditional bedding materials. The light surface peat that is aggregated while mining the deeper layers of peatlands for energy purposes has also been used widely as animal bedding. The future of all peat utilization remains uncertain because of ecological concerns (7) and hence changing environmental legislation.

Different bedding materials vary in their qualities regarding their abilities to absorb moisture and ammonia and contain variable concentrations of inhalable and respirable particles, along with microbes and endotoxins (8–12). Due to the high prevalence of respiratory diseases, especially equine asthma, amongst horses worldwide and the importance of air quality on the pathophysiology of these diseases, bedding choice should be considered fundamental. Scarce scientific literature exists focusing on the specific effects of bedding material on equine airway inflammation (12–14), as most of the research on the matter has covered various combinations of feed and bedding material (1, 5, 8).

Peat bedding has empirically been considered a good choice for horses affected with respiratory disease. Peat has recently been found to be superior compared to wood shavings when equine lower airway cytology was investigated (14). In this previous study (14), peat bedding was suggested to cause a lower neutrophil percentage in the lower airway of healthy horses compared to wood shavings. However, the composition of peat may vary depending on its origin, and various peat storage methods could have further negative effects on its hygienic quality.

The aim of our current study was to compare different bedding materials to the peat bedding that was used in our first study (14), and to further investigate the effects of these bedding materials on clinical respiratory variables, and on the tracheal mucus score and lower respiratory tract cytology of healthy horses. We hypothesized that peat induces less airway inflammation than straw pellets or wood pellets.

## Materials and Methods

### Study Design

A prospective, experimental study was carried out in Ypäjä, southwestern Finland during winter 2018–2019. In the study, we compared the effects of four bedding materials on equine lower airway inflammation in a clinical setting, where each horse acted as its own control. Each investigated bedding material was used for 35 consecutive days in the following order: baled peat (Peat 2), wood pellet, straw pellet, and loosely stored peat (Peat 3). Wood pellet, straw pellet, and Peat 3 were each compared to Peat 2. This study was conducted as a continuum of another study (14). The Peat 2 period in the current study is referred to as Peat 2 period in the earlier study also (14).

### Animals and Management

A total of 32 adult, clinically healthy research and riding school horses owned by Ypäjä Equine College and Natural Resources Institute Finland were studied. The experimental procedures were approved by the Finnish National Experimental Animal Committee. The horses performed their normal riding school routines during the study, excluding the days when airway sampling was conducted. The horses were housed indoors for approximately 18 h per day and ridden in an indoor riding arena (2–3 h/day). The horses spent the remaining 3–4 h of the day outside on sand paddocks.

The horses were housed in a stable in individual boxes (3 m × 3 m), with box doors opening into common indoor corridors (width 2.8 m). Stable room height was 3.5 m, and the boxes were situated along two parallel aisles with a common airspace. Stable ventilation was mechanically forced with an extractor technique, and the stable doors were kept open as much as possible during the daytime, depending on weather conditions. The stables were manually cleaned daily, mainly when the horses were outside. All feces and wet materials were removed from the box, and a similar quantity of new bedding material was added to maintain an approximately 10-cm bedding depth.

The horses were fed haylage (dry matter 73%), wrapped into large round bales using stretch film, and pelleted compound feed (dry matter 88%) from the same provider and manufacturer over the entire study period. The haylage fulfilled the criteria for high-quality haylage (15). It was fed from the box floor thrice per day: approximately at 6 am, at noon, and at 6 pm. Each horse consumed ca. 8–10 kg of haylage and 1–2.5 kg of pelleted feed per day based on individual needs. The clinical status of the horses was monitored on a daily basis by the stable staff and equine college students working with the horses by recording whether any cough and/or nasal discharge was present.

### Bedding Materials

Bedding materials were stored in a barn, separate from the main stable, during the experiment. The bedding materials were transported to the barn just before each period. The milled lightly decomposed sphagnum peat (Peat 2) (40% moisture, pH 4.0–4.8) was manufactured for horse bedding (Vapo Group, Vantaa, Finland). The maximum granule diameter of this product was 50 mm. It was packed in plastic-covered 85-L bales. The wood pellets were made of spruce and pine and packed in plastic-covered 1,000-kg bags (Fortum Horse Power, Espoo, Finland). Each wood pellet was 8 mm in diameter. The straw pellets were made of wheat straw and packed in plastic-covered 500-kg bags (Fortum Horse Power, Espoo, Finland). Each straw pellet was 8 mm in diameter. The second sphagnum peat (Peat 3) (35–44% moisture, pH 4.0) was also manufactured for animal bedding (Vapo Group, Vantaa, Finland), but unlike Peat 2, this product was delivered to the stable on an open truck and stored as a large, loose stack inside a barn. According to the manufacturer, Peat 3 is milled, unsifted, lightly decomposed peat, which may contain coincidental larger pieces of wood or other organic materials. The maximum granule diameter of this product was 100 mm.

### Airway Sampling

The airway sampling and the processing and analyses of the samples were performed similarly as described in our previous paper (14). Each horse was examined, and samples were taken on days 34 or 35 of each bedding period. The examination included a clinical examination with cardiothoracic auscultation, measurement of heart rate and respiratory rate, measurement of rectal temperature, palpation of submandibular lymph nodes, and inspection for the presence of nasal secretions. Horses were examined and sampled in their own boxes.

After clinical examination, the animals were sedated with intravenous detomidine (0.006–0.02 mg/kg, Domosedan vet inj 10 mg/mL, Orion Corporation, Espoo, Finland) and butorphanol (0.006–0.02 mg/kg, Butordol 10 mg/mL inj, Intervet International, Boxmeer, Netherlands). The airways were examined with a fiber-optic video-endoscope (Pentax EG-2940K, 10 mm × 100 cm), and the clinical findings were recorded by a single experienced person (JM) using a tracheal mucus score from 0 to 5 (16).

Tracheal wash (TW) aspirate samples were obtained with a disposable single lumen catheter (2.3 mm × 220 cm Equivet Endoscope flushing catheter, Kruuse) via an endoscope working channel using 20 mL of saline.

Bronchoalveolar lavage fluid (BALF) samples were obtained using a soft rubber tube (10 mm × 240 cm Equivet B.A.L. catheter with a balloon (10 mL), Kruuse) using a blind technique. Local anesthesia (40 mL of 1% lidocaine diluted with saline, Lidocain 20 mg/mL inj, Orion Corporation, Espoo, Finland) was administered prior to 300 mL of physiological saline (room temperature), which was injected in one single volume followed by immediate manual aspiration. Samples were considered adequate when a foamy surfactant layer was detected. The BALF samples were immediately placed in water mixed with ice and submitted to laboratory processing within 1 h.

### Sample Processing and Analyses

Tracheal wash samples were prepared for differential cell counts by centrifugation and subsequent smear of the cell pellet on a slide. Pooled BALF samples were filtered through a 1-layer cotton gauze and the fluid volume was recorded. The BALF cell count was determined using trypan blue stain (1:1), after which the sample was cytocentrifuged (Thermo Scientific Cytospin 4 centrifuge; Thermo Fisher Scientific, Waltham, MA, USA). All slides were stained with May—Grünwald—Giemsa stain.

300 Cells for TW and 400 Cells for BALF Were Counted Under Light Microscopy at 400× Magnification by a Single Blinded Experienced Observer, and Determined Differential Counts for Macrophages, Lymphocytes, Neutrophils, Eosinophils, Mast Cells, and Epithelial Cells. The Results Were Expressed as a Percentage of the Total Cells.

### Statistical Analyses

The sample size was calculated (http://nrs.harvard.edu/urn-3:HUL.InstRepos:8160851) with BALF neutrophil percentage as a primary outcome in a cross-over design (2-tailed, power 0.8, significance 0.05, standard deviation 2, estimated difference in means 2, estimated group size 20 horses). The remaining statistical analyses were performed using IBM SPSS statistics system for Windows.

If a certain cytology variable was unmeasurably low, the value was imputed as half of the lowest measured proportion. The Shapiro–Wilks test was used to evaluate the normality of data distributions. Differences in respiratory rate and mucus score were calculated with the Wilcoxon Signed Rank Test. Standard data transformations (log, square root) were used to normalize the distributions of the cytology variables. The differences between the periods in the clinical and cytology variables were analyzed with a linear mixed-effects model, where period was used as the sole fixed effect and horse as the random effect. Pairwise differences (with 95% CI) were estimated from the models. Values were considered significant at *P* < 0.05.

### Monitoring Stable Air Quality

The data concerning stable air quality during the experiment is published as Supplementary Material.

## Results

### Animals

Thirty-two adult (mean age 11.8 years, range 4–18 years) healthy riding school horses were included in the study. The composition of the study population was 19 Finnhorses and 13 Warmbloods, with an equal number of mares (*n* = 16) and geldings (*n* = 16). All horses remained free from signs of respiratory disease, including cough, during the entire study period.

### Tracheal Mucus Score and Respiratory Rate

**Figure 1:**
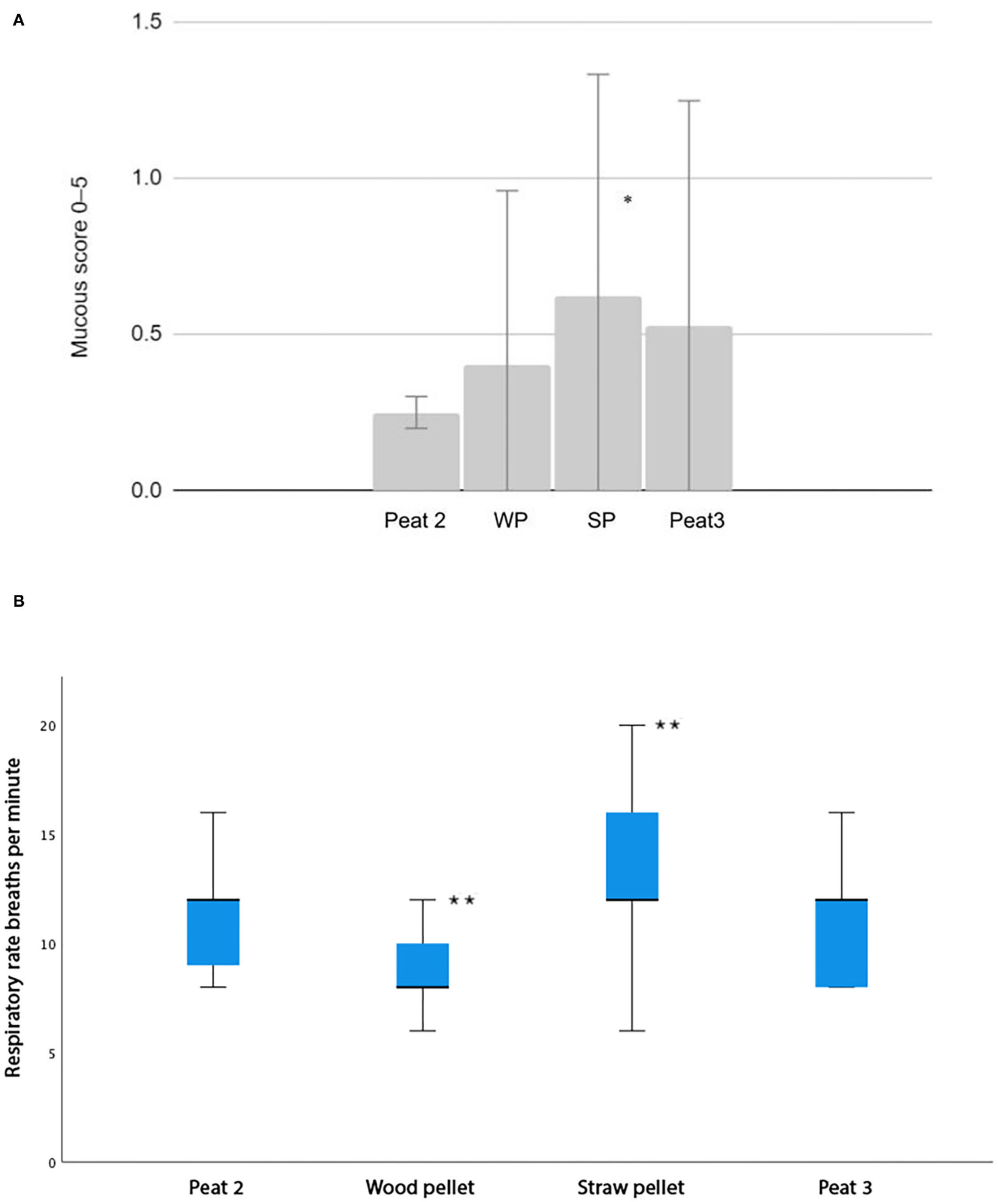
**(A)** A column chart depicting average mucous scores for each bedding material compared to Peat 2. The whiskers represent the standard deviation. WP, Wood pellet, SP, Straw Pellet. Significant differences between groups are indicated as follows: * indicates *p* < 0.05. **(B)** A box plot depicting average respiratory rates as breaths per minute for each bedding material compared to Peat 2. Each box represents the IQR (i.e., 25–75th percentiles), the horizontal line in each box represents the median, the whiskers represent the range. Significant differences between groups are indicated as follows: ** indicates *p* < 0.05.

The tracheal mucus scores (*P* = 0.014; Figure 1A) and respiratory rates (*P* = 0.026; Figure 1B) were higher during the straw pellet period compared to the Peat 2 period. The mucous scores of all the bedding materials remained low, with the average mucous score <1.The respiratory rate was lower during the wood pellet period compared to the Peat 2 period (*P* = 0.004; Figure 1B). For all bedding materials, the average respiratory rates remained in the normal range of 8–15 breaths per minute.

### Cytology

**Figure 2:**
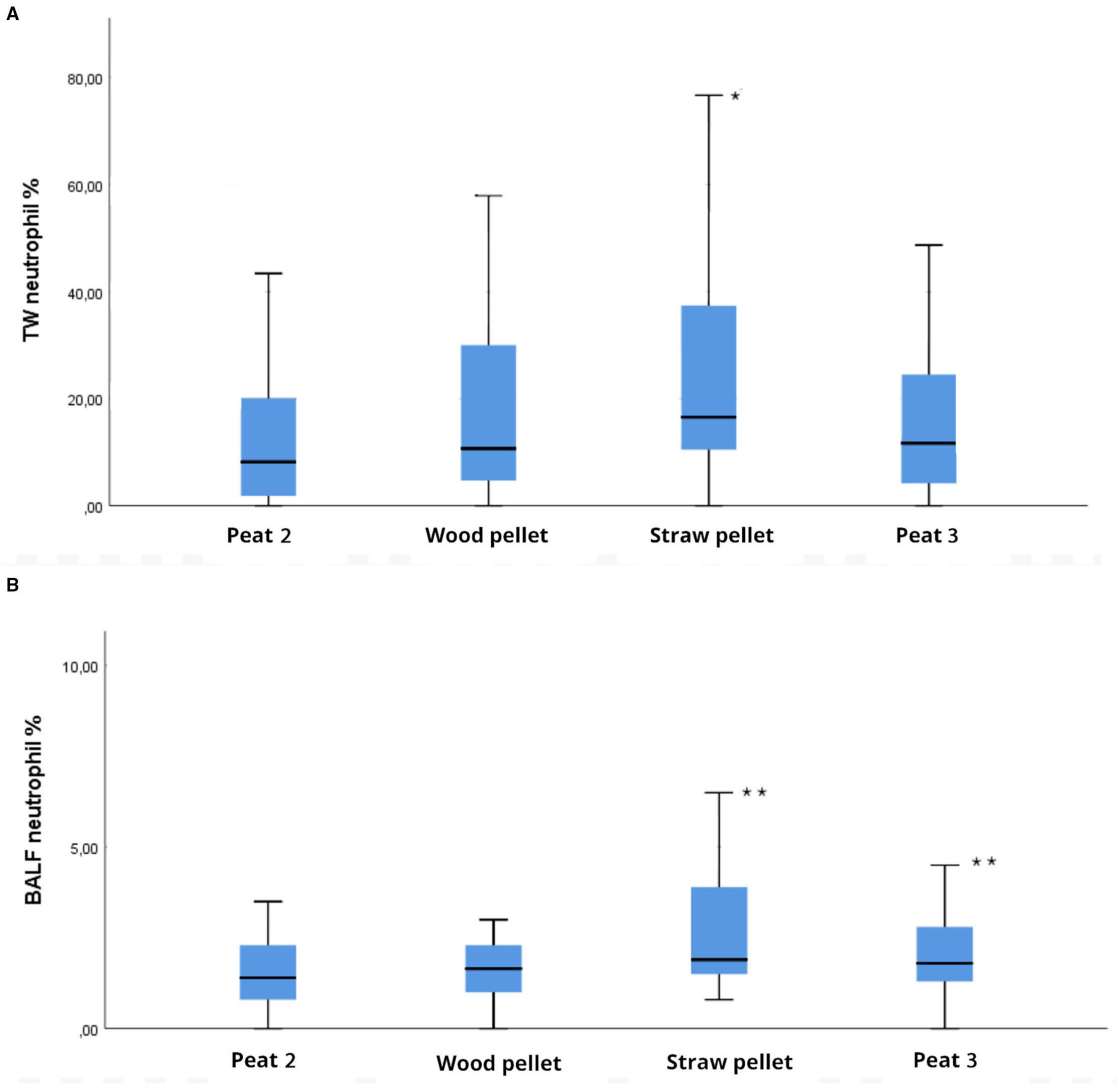
Box plot depicting differences between groups compared to Peat 2 in **(A)** tracheal wash (TW) neutrophil percentages, **(B)** bronchoalveolar lavage fluid (BALF) neutrophil percentages of 32 horses housed on different bedding materials after each bedding material (Peat 2, Wood pellet, Straw pellet, Peat 3). Each box represents the IQR (i.e., 25–75th percentiles), the horizontal line in each box represents the median, the whiskers represent the range. Significant differences between groups compared to Peat 2 are indicated as follows: in **(A)** * indicates *P* < 0.001; in **(B)** ** indicates *P* < 0.05.

The TW neutrophil percentage during the straw pellet period was higher compared to the Peat 2 period (*P* = 0.0003; Figure 2A). We observed no difference between the wood pellet period or the Peat 3 period compared to the Peat 2 period in TW neutrophil percentages (Figure 2A).

The average retrieved volume of BALF for all the sampling points was 149 ml (48–236 ml). The BALF neutrophil percentage was higher during the straw pellet period compared to the Peat 2 period (*P* = 0.005; Figure 2B). The BALF neutrophil percentage was higher during the Peat 3 period compared to the Peat 2 period (*P* = 0.04; Figure 2B). The BALF neutrophil percentage of the wood pellet period did not differ from the Peat 2 period. There was no difference between the bedding materials in terms of BALF mast cell or eosinophil percentages. The proportions of the remaining TW and BALF cell types are presented in Table 1.

**Table 1:** Summary of cytology results (TW, BALF).

	**Peat 2**	**Wood pellet**	**Straw pellet**	**Peat 3**
**TW cell type %**				
Macrophages	71.3 (15–98)	75 (13.7–96.7)	60.7 (11–93.4)	71.5 (0.7–95.7)
Lymphocytes	0.7 (0–2.7)	0.4 (0–52)	1.7 (0–60)	0.9 (0–11)
Eosinophils	0 (0–4.7)	0.1 (0–0.7)	0.4 (0–3.4)	0.3 (0–2.7)
Epithelial cells	8.6 (1–82.4)	3.4 (0–41.4)	4.1 (0–87.7)	4.9 (0–48.7)
**BALF cell type %**				
Macrophages	44.5 (27–69.8)	46.15 (29–73.3)	41.2 (20.8–63.3)	38.7 (5.3–71.5)
Lymphocytes	52.8 (25.5–71.8)	47.7 (18.5–66.8)	54.8 (30.5–72.8)	55.7 (0.8–82.5)
Eosinophils	0 (0–0.3)	0.1 (0–1.5)	0.1 (0–1.0)	0.1 (0–0.8)
Mast cells	0 (0–0.7)	0 (0–0.4)	0 (0–1.3)	0 (0–0.7)
Epithelial cells	0 (0–0.3)	0 (0–0.8)	0.3 (0–1.8)	0.5 (0–1.8)
**BALF total nucleated cell count**	224 (70–380)	237 (90–820)	252 (100–440)	301 (60–1370)

*Numeric values are presented as medians with range in brackets*.

## Discussion

This study compares the effects of peat, wood pellet, and straw pellet as bedding materials regarding the lower airway cytology of healthy horses for the first time. Other bedding materials were compared to peat. In the present study, a higher percentages of neutrophils were detected in both TW and BALF after the straw pellet period compared to the Peat 2 (baled peat) period. The BALF neutrophil percentage was higher during the Peat 3 period compared to the Peat 2 period, but there was no difference in TW neutrophil percentages between these bedding materials. Our hypothesis, formed from practical experience of peat being a good choice for horse bedding when lower airway inflammation is being considered, is supported by research from the last few years (13, 14). A previous study (14), performed with the same horses as those in the current study, concluded that the lower airway samples had higher neutrophil percentages when horses were kept on wood shavings compared to peat.

The tracheal mucus score and respiratory rate were higher during the straw pellet period compared to the Peat 2 period. These mild changes in clinical findings are well in line with the mild to moderate increases in neutrophil percentages in the lower airway cytology. Only such subtle differences between the bedding materials were expected, as all the horses in the study were reportedly healthy from their airways both before and during the study. The respiratory rate was lower during the wood pellet period compared to the Peat 2 period, albeit we detected no difference in these two bedding materials when the airway cytology results were compared. As evident, these findings (clinical findings vs. increased lower airway neutrophil percentages) were inconsistent. For example, the BALF neutrophil percentage was higher during the Peat 3 period compared to the Peat 2 period while there was no difference in the tracheal mucus score or respiratory rate between these bedding materials. For all bedding materials, the mucous scores remained low and the average respiratory rates remained within their normal ranges.

Despite the commonly encountered negative effects of straw on stable air quality (9) and on equine lower airway inflammation (17), which have been acknowledged for years, straw harvested and stored in traditional ways is still widely used as horse bedding because of its good availability and the comfort it provides to horses (18). The literature on straw pellets is scarce. They are produced by heating and removing dust from the pellet surface (19). These measures could reduce the microbial air contamination and dustiness compared to straw, making the pellet form possibly more suitable for equine bedding compared to straw. In a study by Fleming et al. (2), straw pellets were associated with lowest particle concentrations (particle size smaller than 10 μm) in stable air compared to wood shavings and straw. In another study by the same author (11), straw pellet was found to be superior in its ammonia-binding capacity compared to wheat straw, wood shavings, hemp, linen, and paper cuttings. However, in our study, straw pellets caused the highest neutrophil percentages in both the TW and BALF samples. Once again, comparing the results between studies is challenging for many reasons. The bedding materials compared in Fleming's experiments (2, 11) and ours differed, as did the methods used: Fleming's studies focused on respirable air quality while our study examined airway cytology. Anyhow, the contradiction between these results emphasizes the role of airway sampling when assessing the effects of various bedding materials or forage on stable air quality. The cascade of sterile inflammation in equine lower airways is affected by many external factors: the respirable fraction of dust; the fungi, mold, and bacteria this fraction contains; the endotoxins and 1,3-β-glucans of the aforementioned; other respiratory irritants, such as ammonia and cold air, along with the cumulative effect of stabling and breathing in all these impurities long term (4, 17). These factors all contribute and interact with each other in ways that are evidently still beyond our current understanding. The specific features of bedding materials can be analyzed exhaustively, but due to the complexity of the matter—further complicated by the marked effects of forage on the air quality—investigating the airway response to all this may offer a better perspective.

In this study, we found no significant difference between the baled peat (Peat 2) and the wood pellets, whereas our earlier study (14) concluded that wood shavings were associated with increased neutrophil percentages in the lower airway samples compared to peat. Two pelleted products (straw pellet, wood pellet) were investigated in our current study. As already discussed, straw pellet caused the highest neutrophil percentages in both airway sample types and also caused significantly higher neutrophil percentages compared to Peat 2. The pellet form of the bedding material does not therefore seem to be the key element in reducing the bedding material's negative effects on equine airways: based on our results, wood pellet was a good option and straw pellet the worst option from this perspective. A Polish study (12) compared crushed wood pellets to straw and a mixture of peat and wood shavings. They found crushed wood pellets to cause more respirable dust than straw or a peat–wood shavings mixture. On the other hand, crushed wood pellets caused less microbial air contamination than the other alternatives. For these three options, crushed wood pellet had the lowest endoscopic score of the tracheal examinations despite its high respirable dust content. Lower airway cytology was not performed in this study, which makes it difficult to assess the true degree of lower airway inflammation. Higher respirable dust concentrations have previously been shown to correlate with the degree of airway inflammation (20, 21), but as discussed previously, the role of other respiratory irritants besides dust levels must be considered.

Measuring the various size fractions of dust has been a common way to evaluate the effects of bedding materials on stable air and thus on equine respiratory health. Dust particle size caused by the bedding material is one crucial factor (22) determining how much dust actually reaches the small airways. With peat, particles are mostly larger than 10 μm in diameter (23). For this reason, most of the peat dust can be assumed to be deposited in the proximal airways, leaving only slight alveolar-level contamination. In our previous article (14), we speculated that this could be one key element explaining peat's superiority compared to wood shavings. The difference in BALF neutrophil percentages between Peat 2 and Peat 3, which we present in the current study, is of special interest, as both products are marketed for and used as horse bedding material. Regarding BALF neutrophil proportions, we found a significant difference between loose peat (Peat 3) and baled peat (Peat 2) in favor of the baled peat. The dustiness and hygienic quality of peat can be somewhat unpredictable (9, 24), and these factors are not routinely analyzed from products on the market, making practical comparisons of the products prior to purchase impossible. The dust particle size of the two peat products used in our study should be similar, as the products are made of the same light surface peat, with the difference that Peat 2 was sifted and thus had a smaller macroscopic grain size. On the other hand, the coarser constitution of Peat 3 could lead to more microbial contamination, as it contains larger pieces of less-decomposed organic material.

Furthermore, the way in which loose peat (Peat 3) is stored may affect its hygienic quality. When loose peat is stored in large stacks, conditions may become favorable for heating and subsequent aerobic microbial overgrowth, especially if peat moisture is very variable within the stack (25). Overheated peat becomes “overdry” and paradoxically loses its ability to absorb moisture. Peat has been shown to contain pathogens such as mycobacteria (26) and *Aspergillus fumigatus* (24). Warming the peat to 30°C increases its fungi levels (24). Peat stack heating can be assessed by monitoring its temperature either subjectively by hand or using special thermometers. In our experiment, stack temperature was not monitored, and to our knowledge it is not a common practice in stables.

The effect of long stabling (27) and winter weather conditions in the Northern Hemisphere (28) have been suggested to cause increasing neutrophil percentages in the lower airways of healthy horses. In Hansen's study (28), mild winter weather conditions with lower temperatures, fewer minutes of sunlight, and a higher humidity percent were associated with a significantly higher BALF neutrophil percentage and a higher mucus score in stabled clinically healthy horses. This phenomenon was not detected in our study. As the experiment was conducted between December and May, the horses had been stabled for 4 months before the first sampling was performed. In contrast to Hansen's study (28), where a significantly higher percentage of neutrophils and a higher tracheal mucus score were found during November compared to May, the highest neutrophil percentages in our study were found after the straw pellet period, which ended at the beginning of April. Indoor air humidity percentages in our study were also higher during the Peat 2 and Wood shavings periods than with the Straw pellet and Peat 3 periods (Supplementary Material). These rather conflicting findings between the studies once again emphasize the myriad of factors influencing equine lower airway inflammation.

We observed some differences between the TW and BALF cytology results. Straw pellet was the only bedding material causing increased neutrophil percentages in both TW and BALF compared to Peat 2, whereas Peat 3 caused an increase in neutrophil percentage only in BALF, not in TW in comparison to Peat 2. The median neutrophil percentage in TW after the straw pellet period exceeded the upper reference range for this cell type [<20% (17)], whereas the median neutrophil percentage in BALF remained within normal limits [<5% (17)]. In previous literature, the study results differ in their agreement between TW and BALF cytology results. Two quite recent studies from Scandinavia showed good agreement between these sample types in the neutrophil percentages (28, 29), although BALF has traditionally been preferred over TW in diagnosing lower airway inflammation in horses (17, 21). BALF results showed better consistency in our present study. It is still unclear if all the phenotypes of equine asthma even can be diagnosed using current BALF methodology and cut-off values for inflammatory cell proportions (30). There can be a fine line between a healthy individual and a horse suffering from a form of mild equine asthma. Even more detailed clinical scoring systems than the one that was used in our study have failed to differentiate healthy horses from the ones affected with mild equine asthma (30). Lung function testing would offer more information about the airway hyperresponsiveness than mere BALF in some cases (31). We were not able to perform lung function tests to the horses used in this study for practical reasons, as the horses were examined in their own stable.

The study design has a few inherent weaknesses, which may cause some bias in the results. Airway neutrophilia caused by exogenous triggers is evident in asthmatic horses 6 h after allergen exposure (32) and may persist for some weeks after a change of environment and remission of clinical signs (33). This phenomenon could have caused some carry-over effect on the airway cytology results of the following bedding material period, as our study setting did not include wash-out periods. As the horses in our study had healthy airways, these types of patterns may not apply. In a recent study comparing the effects of hay and haylage feeding on the BALF of healthy Standardbred horses, haylage caused a decrease in BALF neutrophil percentage compared to the baseline within 2 weeks of use (34). These results may be more comparable to ours, as healthy horses were used in both studies. Longer exposure times than the 35 days we used for each bedding material could have induced more marked differences between the bedding materials. As the indoor housing season length is limited, and we wanted to investigate as many bedding materials as possible, longer periods for each bedding material or wash-out periods between them were not feasible. In our previous study, we saw a decrease in TW and BALF neutrophil percentages with the reference bedding material (baled peat), after the increase seen with wood shavings. This would suggest that the 35 day exposure to the bedding material is long enough to provide reversal of previously altered airway cytology related to bedding exposure. Not all changes in daily horse management could be avoided because the horses were not experimental animals kept solely for research purposes. The horses were handled and fed by the same professionals during the study, which probably minimized variation. Also, we performed the airway sampling at the same time of the day for each period to avoid variation in the stable air quality caused by stable cleaning or movement of the horses. The examiner assigning tracheal mucous score was not blinded to the type of bedding material and this could cause possible bias in this grading.

The focus of our study was on how bedding materials affect the lower airway cytology of horses. In the Supplementary Material, we provide data on air quality monitoring during the experiment. Unfortunately, dust level monitoring was inconsistent, and the respirable fraction of the dust was not measured. The average indoor temperatures of the stable remained within the target temperature range (8–12°C) for horse stables in Finland (35), but the stable air humidity was much higher compared to the target value (50–55%) for all investigated periods. The indoor conditions were similar in the earlier study (14) performed in the same stable. The weather in Northern Europe, and thus the conditions in the stables during the indoor housing season differ from conditions in more southern countries. The presence of airborne dust particles is influenced by temperature and air humidity, and for this reason our results may not necessarily be applicable to more temperate regions of the world.

## Conclusions

In conclusion, baled peat bedding was associated with a smaller neutrophil percentage in the lower airway of healthy horses compared to straw pellets and loosely stored peat. We observed no significant difference between baled peat and wood pellets in the inflammatory cell proportions. The results of our study support the initial hypotheses of peat being a suitable option as a horse stable bedding material and it being superior to straw pellets when considering equine airway health. The results are in line with results from our previous study, which compared the same baled peat to wood shavings, in favor of peat. The manufacturing process and storage of peat bedding may play a role in its hygiene level, as the baled peat product was superior to the loosely stored peat. The information gained from this study may assist veterinarians and horse owners in selecting bedding materials, especially for horses suffering from respiratory diseases. More research on the effect of bedding materials in horses with various respiratory conditions are warranted. As the use of peat may not be a long-term solution due to its future availability, focus should be turned to other bedding materials that share similar properties with peat.

## Data Availability Statement

The raw data supporting the conclusions of this article will be made available by the authors, without undue reservation.

## Ethics Statement

The animal study was reviewed and approved by the Finnish National Experimental Animal Committee. Written informed consent was obtained from the owners for the participation of their animals in this study.

## Author Contributions

JM, MS, and AM contributed to the following steps of the manuscript preparation process: generating the hypothesis and designing the experiment, organizing and conducting the experiment, interpreting and analyzing the results, and writing and revising the manuscript. NK contributed to generating the hypothesis, designing the experiment, and organizing and conducting the experiment. MR contributed to generating the hypothesis and designing the experiment. MN contributed to organizing and conducting the experiment and interpreting and analyzing the results. All authors contributed to the article and approved the submitted version.

## Funding

The bedding materials were sponsored by Vapo Group and Fortum Horse Power.

## Conflict of Interest

The authors declare that the research was conducted in the absence of any commercial or financial relationships that could be construed as a potential conflict of interest.

## Publisher's Note

All claims expressed in this article are solely those of the authors and do not necessarily represent those of their affiliated organizations, or those of the publisher, the editors and the reviewers. Any product that may be evaluated in this article, or claim that may be made by its manufacturer, is not guaranteed or endorsed by the publisher.
