# Supplementary material for: Effects of Bedding Material on Equine Lower Airway Inflammation: A Comparison of Two Peat Beddings, Wood Pellet, and Straw Pellet
Source: Front Vet Sci. 2021 Dec 17;8:799645. doi: 10.3389/fvets.2021.799645 (PMC8718510; doi:10.3389/fvets.2021.799645)
Supplement: Supplementary file 1 [file Table_1.pdf]

# *Supplementary Material*

| | **Peat 2** | **Wood pellet** | **Straw pellet** | **Peat 3** |
|---|---|---|---|---|
| Stable dust (mg/day), mean of 3 containers (min–max) | 5.7 (2.3–10.2) | 3.3 (2.7–4.6) | 2.8 (2.2–3.1) | 2.6 (2.3–2.9) |
| Amount of ammonia in stable air (ppm) | 0 | 0.25 | 0.25 | — |
| Indoor temperature (°C) | 11.0 (7–13) | 8.9 (5–12) | 11.0 (7–15) | 10.5 (8–13) |
| Indoor air humidity (%) | 86.2 (75–94) | 81.5 (74–90) | 73.6 (63–85) | 70.0 (60–73) |
| Outdoor temperature (°C) | 4.2 (-5.9–7.9) | -11.2 (-19.7–1.4) | 1.5 (-9.6–6.4) | 5.5 (-2.3–13.7) |

Total stable dust was measured by collecting dust with open plastic containers (13-cm deep containers, surface area 188.8 cm2) placed at a height of 2.5 m at three locations within the stable. The quantity of dust collected in the containers was weighed and expressed as mg/day.
